# Habitat-Dependent Ecological Differentiation of Soil and Water Microbiomes in High-Altitude Alpine Meadow Ecosystems on the Qinghai–Tibetan Plateau

**DOI:** 10.3390/microorganisms14071489

**Published:** 2026-07-08

**Authors:** Chen Duan, Dongyang Wang, Lang Tan, Qi Wang, Zhankun Tan, Yanfen Cheng

**Affiliations:** 1 Laboratory of Gastrointestinal Microbiology, National Center for International Research on Animal Gut Nutrition, Nanjing Agricultural University, Nanjing 210095, China; 2 Key Laboratory of Plateau Grazing Animal Nutrition and Feed Science of Qinghai Province, Qinghai University, Xining 810016, China; 3 College of Animal Science, Xizang Agricultural and Animal Husbandry University, Linzhi 860000, China; 4 State Key Laboratory of Grassland Agro-Ecosystems, Center for Grassland Microbiome, College of Pastoral Agriculture Science and Technology, Lanzhou University, Lanzhou 730000, China

**Keywords:** Qinghai–Tibetan Plateau, alpine meadow ecosystems, soil and water microbiomes, community assembly, antibiotic resistance genes

## Abstract

High-altitude ecosystems are characterized by extreme environmental conditions that strongly influence microbial community structure and function. However, whether soil and water microbiomes exhibit similar ecological responses to environmental variation in alpine meadow ecosystems on the Qinghai–Tibetan Plateau remains poorly understood. Here, we combined 16S rRNA gene amplicon sequencing and metagenomic sequencing to compare soil and water microbiomes across two regions (LZ and NQ) with distinct physicochemical profiles. Environmental heterogeneity was more pronounced in water habitats, where all measured parameters (pH, total nitrogen, total organic carbon, and chemical oxygen demand) varied significantly between sites (*p* < 0.001). Correspondingly, water microbiomes exhibited greater regional differentiation than soil microbiomes, evidenced by stronger beta-diversity separation (PERMANOVA, R^2^ = 0.667 vs. 0.376) and a lower proportion of shared ASVs (65.3% vs. 97.2%). Ecological assembly analysis revealed a sharp contrast: water communities were primarily governed by deterministic processes (accounting for >80% of assembly, with heterogeneous selection as the dominant driver), whereas soil microbiomes were dominated by stochastic processes (>50%). Furthermore, water microbiomes underwent more intense network restructuring, with interaction complexity increasing significantly from 70 nodes and 268 edges in the LZ region to 130 nodes and 577 edges in the NQ region, whereas soil networks remained relatively stable (146 nodes/368 edges to 128 nodes/391 edges). Functional profiling further indicated broader regional redistribution in water compared to the relatively conserved functional framework of soil communities. Resistome analysis identified distinct ARG structures between habitats while revealing 25 overlapping categories, suggesting potential ecological connectivity. Collectively, our findings demonstrate that water microbiomes are more sensitive to regional environmental variation than soil microbiomes, with aquatic communities responding through deterministic restructuring and heightened interaction complexity. These results provide quantitative evidence that high-altitude soil and water microbiomes adopt distinct ecological strategies, offering new insights into the mechanisms governing microbial adaptation and antibiotic resistance distribution.

## Simple Summary

High-altitude ecosystems on the Qinghai–Tibetan Plateau are exposed to harsh environmental conditions, including low temperatures, strong ultraviolet radiation, and low oxygen availability. Microorganisms play essential roles in maintaining ecosystem functions in these environments. However, it remains unclear whether soil and water microbiomes respond similarly to environmental variation in alpine meadow ecosystems on the Qinghai–Tibetan Plateau. In this study, we compared microbial communities in soil and water habitats from two high-altitude alpine meadow regions with contrasting elevations on the Qinghai–Tibetan Plateau. We investigated microbial diversity, community composition, ecological interactions, assembly processes, functional potential, and antibiotic resistance genes. Our results showed that soil and water microbiomes responded differently to regional environmental variation. Water microbiomes exhibited stronger community differentiation between regions, accompanied by more pronounced shifts in taxonomic composition, ecological network structure, community assembly processes, and functional profiles. In contrast, soil microbiomes maintained a relatively stable core community despite environmental differences. We also found that soil and water habitats shared a substantial proportion of antibiotic resistance genes, suggesting potential ecological connectivity between terrestrial and aquatic microbial communities. These results enhance our understanding of habitat-associated differences in microbial communities in alpine meadow ecosystems on the Qinghai–Tibetan Plateau, highlighting how environmental variation between soil and water shapes taxonomic composition, ecological network structure, functional potential, and antibiotic resistance gene profiles.

## 1. Introduction

The Qinghai–Tibetan Plateau (QTP), widely recognized as the “Third Pole of the Earth,” represents the highest and largest alpine plateau globally [1]. This region is characterized by extreme environmental conditions, including low temperatures, intense ultraviolet radiation, and marked environmental heterogeneity [2,3]. These harsh conditions impose significant selective pressures on resident microbial communities [4], establishing the QTP as an ideal natural laboratory for investigating microbial adaptation and ecological processes under environmental stress [5,6].

Alpine meadow ecosystems dominate the QTP landscape, where soil and water environments function as tightly coupled components that regulate nutrient cycling, organic matter turnover, and broader biogeochemical processes [7,8]. Microorganisms are pivotal in mediating these ecological functions and are highly sensitive to habitat properties [9,10].

While environmental gradients are known to strongly shape microbial diversity and community composition across the QTP, previous research has largely been confined to soil ecosystems [11,12]. Consequently, most existing literature treats terrestrial and aquatic habitats in isolation, with limited attention paid to their coordinated ecological responses [13,14]. Although freshwater habitats serve as vital interfaces linking the terrestrial–aquatic continuum [15], their microbial communities are rarely examined in direct comparison with adjacent soils [16]. As a result, how soil and water microbiomes jointly respond to high-altitude environmental variation within alpine meadow ecosystems remains poorly understood. This study aims to fill this knowledge gap by exploring the distinct ecological strategies and functional adaptations of soil and water microbiomes in these high-altitude environments.

Beyond taxonomic and functional variation, environmental microbiomes also harbor diverse antibiotic resistance genes (ARGs), collectively referred to as the environmental resistome [17]. Although ARGs have traditionally been studied from a public health perspective, increasing evidence suggests that they represent intrinsic components of natural microbial communities, contributing to microbial competition, environmental adaptation, and ecosystem resilience [18,19]. Soil is widely recognized as a major natural reservoir of ARGs [18], whereas aquatic environments may facilitate the transport and dissemination of resistance determinants across connected habitats [20]. Despite their ecological significance, little is known about how resistome profiles differ between adjacent soil and water habitats in alpine meadow ecosystems of the Qinghai–Tibetan Plateau.

To address these knowledge gaps, we investigated soil and water microbiomes from two alpine meadow regions on the Qinghai–Tibetan Plateau that differ markedly in elevation and environmental conditions (Nyingchi, 2900 m; Nagqu, 4700 m). Using 16S rRNA gene amplicon sequencing and shotgun metagenomic sequencing, we characterized microbial community composition, ecological interactions, community assembly processes, functional potential, and antibiotic resistance profiles across habitats. We hypothesized that the contrasting physicochemical characteristics of soil and water habitats would lead to distinct microbial ecological responses to regional environmental variation. Specifically, we expected that water microbiomes, owing to their greater sensitivity to environmental change, would exhibit stronger taxonomic, ecological, and functional differentiation than soil microbiomes. We further anticipated that soil and water microbiomes would harbor distinct resistome compositions, with the potential for cross-habitat exchange of antibiotic resistance genes.

## 2. Materials and Methods

### 2.1. Study Area Description

The study was conducted in two geographically distinct high-altitude alpine meadow regions on the Qinghai–Tibetan Plateau, Nyingchi and Nagqu. These regions differ markedly in climatic and environmental conditions and are representative of alpine meadow ecosystems within this plateau. A total of six sampling sites were selected, with three sites in each region. At each site, paired soil and water samples were collected from natural alpine meadow habitats under traditional grazing conditions. These sites provide a representative framework for comparing soil and water microbiomes across contrasting environmental gradients. Detailed information on geographical coordinates and elevations is provided in Table 1.

### 2.2. Experimental Design

This study was designed to investigate ecological differentiation between soil and water microbiomes in response to regional environmental variation in high-altitude alpine meadow ecosystems on the Qinghai–Tibetan Plateau. Surface soil and water samples were collected from six sampling sites across two regions (Nyingchi and Nagqu) to characterize microbial community composition, functional potential, and antibiotic resistance gene (ARG) profiles. The study design incorporated two habitats (soil and water) and two regions, enabling a paired comparison of habitat-specific and region-specific microbial ecological patterns under contrasting environmental conditions.

### 2.3. Sample Collection

Sampling was conducted during the peak growing season across all study sites. In each alpine meadow, surface soil (0–10 cm depth) and water samples were collected to represent the distinct terrestrial and aquatic compartments of the ecosystem. The study sites are characterized by sandy loam soil, typical of the high-altitude alpine meadow ecosystems on the Qinghai–Tibetan Plateau, often featuring dense root mats and high organic matter accumulation in the surface layer. To minimize micro-scale vertical and spatial heterogeneity—particularly variations in aeration, moisture, and redox gradients—we employed a composite sampling strategy. At each site, soil cores (0–10 cm) and water samples were collected from ten randomly selected locations within the plot. These subsamples were thoroughly homogenized and pooled into one representative composite soil sample and one composite water sample per site. All samples were immediately transported to the laboratory on ice and stored at −80 °C until DNA extraction and downstream analyses.

### 2.4. Determination of Physicochemical Properties of Water

Physicochemical properties of water samples, including pH, total nitrogen, total organic carbon, and chemical oxygen demand, were measured following established protocols. The pH was measured in situ using a handheld water quality analyzer (PTF-001B, Pantian Biotechnology, Xiamen, China). TN and TOC were quantified using a TOC analyzer (multi N/C 3300, Analytik Jena, Jena, Germany). COD was determined using the rapid digestion spectrophotometric method (HJ/T 399–2007) [21].

### 2.5. Determination of Physicochemical Properties of Soil

Soil physicochemical properties, including pH, total nitrogen, total phosphorus, and organic matter, were measured following national standards and published methods. Soil pH was measured at a 1:2.5 (soil:water) ratio using a portable pH meter (PHS-4, REX, Shanghai, China) according to NY/T 1377–2007 [22]. TN was determined using an elemental analyzer (Vario EL III, Elementar, Langenselbold, Germany) following LY/T 1228–2015 [23]. TP was measured using the molybdenum–antimony colorimetric method (HJ 632–2011) [24]. Organic matter was determined using the potassium dichromate oxidation method.

### 2.6. DNA Extraction

Total genomic DNA was extracted from soil and water samples using a standardized protocol provided by a commercial service provider (BGI, Shenzhen, China). Briefly, 100–200 mg (soil) or equivalent filtered biomass (water samples) was subjected to mechanical disruption followed by enzymatic and thermal lysis at 65 °C to ensure efficient cell breakage. DNA was purified using phenol–chloroform–isoamyl alcohol extraction combined with magnetic bead-based cleanup on a KingFisher™ Flex system (Thermo Fisher Scientific, Waltham, MA, USA), with Proteinase K and RNase A treatments applied to remove protein and RNA contaminants. The quality and concentration of the extracted DNA were assessed using a NanoDrop spectrophotometer and a Qubit fluorometer (Thermo Fisher Scientific, Waltham, MA, USA). All DNA samples were stored at −80 °C until downstream sequencing analysis.

### 2.7. 16S rRNA Gene Amplicon Sequencing and Analysis

The V3–V4 region of the bacterial 16S rRNA gene was amplified using primers 338F/806R and sequenced on the DNBSEQ-G400 platform (paired-end 300 bp) after library preparation via rolling circle amplification to generate DNA nanoballs. PCR amplification was performed using high-fidelity polymerase under standard cycling conditions (95 °C denaturation, 56 °C annealing, and 72 °C extension), and amplicons were purified and quality-checked prior to sequencing.

Raw reads were processed through a standardized bioinformatics pipeline, including quality filtering using FastQC (v0.12.1) and fastp (v0.23.4) [25,26], read merging (VSEARCH) [27], and amplicon sequence variant (ASV) inference using UNOISE3 after dereplication and denoising. Taxonomy was assigned against the SILVA v138.1 database using the SINTAX classifier, and non-bacterial sequences were removed. The resulting ASV table was rarefied to an even sequencing depth for downstream analyses.

Microbial diversity was evaluated using alpha and beta diversity metrics, while community interaction patterns were explored via Spearman-based co-occurrence networks constructed with the igraph package [28]. All raw sequencing data were deposited in the NCBI Sequence Read Archive under BioProject PRJNA1463583.

### 2.8. Metagenomic Sequencing and Data Preprocessing

Metagenomic shotgun sequencing was conducted on 47 samples (26 soil and 21 water samples) using the DNBSEQ high-throughput platform (PE150) (BGI, Shenzhen, China). Genomic DNA was randomly fragmented to 200–400 bp using a Covaris ultrasonicator, followed by magnetic bead-based size selection. Sequencing libraries were constructed according to the DNBSEQ standard protocol, including end-repair, 3′-adenylation, adapter ligation with unique indices, PCR amplification, and circularization to generate single-stranded DNA, which was further amplified into DNA nanoballs via rolling circle replication.

Raw reads were processed using fastp (v0.23.4) for adapter removal, quality trimming, and filtering of low-quality or ambiguous reads. Host-derived sequences were removed by aligning high-quality reads against the human (GRCh38.p14) reference genomes using BWA-MEM (v0.7.18) [29], and unmapped reads were retained for downstream assembly and functional analyses.

All metagenomic sequencing data have been deposited in the NCBI Sequence Read Archive under BioProject PRJNA1458465.

### 2.9. Abundance Profiling and Functional Annotation

Functional annotation was performed using a non-redundant gene catalog constructed in a previous study conducted by our group (unpublished data), which was used as the reference database for downstream analyses. EggNOG-mapper [30] was used to assign COG, GO, and KEGG orthology classifications. Metabolic pathways were reconstructed using KofamScan [31] against the KEGG database, while antibiotic resistance genes (ARGs) were identified using the Comprehensive Antibiotic Resistance Database (CARD) [32].

For functional abundance profiling, high-quality reads were mapped to the same reference gene catalog using Salmon [33] in metagenomic mode, and gene abundance was normalized as transcripts per million (TPM) for downstream functional and comparative analyses.

### 2.10. Statistical Analysis

All statistical analyses were conducted using R software (v4.4.1). The normality of data distributions was evaluated using the Shapiro–Wilk test prior to hypothesis testing. Student’s *t*-test was used when data met the assumptions of normality, whereas the Wilcoxon rank-sum test was applied to non-normally distributed datasets. To control the false discovery rate, *p*-values were adjusted using the Benjamini–Hochberg procedure, and statistical significance was determined at an adjusted *p*-value < 0.05. Microbial community dissimilarities were calculated based on Bray–Curtis distances derived from the gene abundance matrix. Variations in community structure among groups were evaluated using permutational multivariate analysis of variance (PERMANOVA) implemented in the adonis2 function of the vegan package with 999 permutations. To quantify microbial community assembly processes, we utilized the ecological modeling framework based on phylogenetic turnover (β-nearest taxon index, βNTI) and taxonomic turnover (Raup–Crick metric, RC_bray_), as described by Stegen et al. [34]. Deterministic processes were identified when |βNTI| > 2. When |βNTI| ≤ 2, community assembly was partitioned into stochastic processes, including dispersal limitation (RC_bray_ > 0.95), homogenizing dispersal (RC_bray_ < −0.95), and undominated/drift processes (|RC_bray_| ≤ 0.95). For LEfSe analysis, the Kruskal–Wallis test was employed to identify features with significant differential abundance, with a significance threshold of *p* < 0.05. Subsequently, a logarithmic linear discriminant analysis (LDA) score threshold of 3.0 was applied to determine the robust biological relevance of these biomarkers.

## 3. Results

### 3.1. Physicochemical Divergence Between Soil and Water Habitats

**Figure 1:**
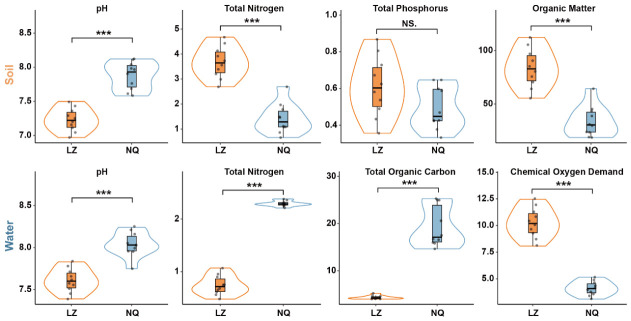
Physicochemical characteristics of soil and water habitats at the study sites. The sample size for each group (LZ and NQ) is *n* = 10. Boxplots overlaid with jittered points represent the distribution of environmental variables, with violin plots showing data density. Top row (Soil): physicochemical properties, including pH, total nitrogen, total phosphorus, and organic matter. Bottom row (Water): physicochemical properties, including pH, total nitrogen, total organic carbon, and chemical oxygen demand. Statistical significance between the LZ and NQ sites was assessed using the Wilcoxon rank-sum test. NS, not significant; *** *p* < 0.001.

The environmental conditions differed significantly between the LZ and NQ sites (Figure 1). A comparative analysis revealed distinct physicochemical profiles for both soil and water habitats. In the soil (Figure 1, Top row), pH, total nitrogen, and organic matter were significantly higher at the NQ site than at the LZ site (*p* < 0.001), while total phosphorus showed no significant variation between regions.

In contrast, the water habitats (Figure 1, Bottom row) exhibited more profound physicochemical divergence; all measured parameters—pH, total nitrogen, total organic carbon, and chemical oxygen demand—varied significantly between sites (*p* < 0.001). Furthermore, a clear habitat-dependent shift was observed for key indices such as pH and total nitrogen, where the absolute values and variability patterns differed markedly between the soil and water habitats. These findings highlight substantial environmental heterogeneity across the high-altitude landscape and provide a reliable basis for distinguishing the physical and chemical constraints inherent to these two contrasting habitats.

### 3.2. Water Microbiomes Respond More Strongly to Regional Environmental Variation Than Soil Microbiomes

**Figure 2:**
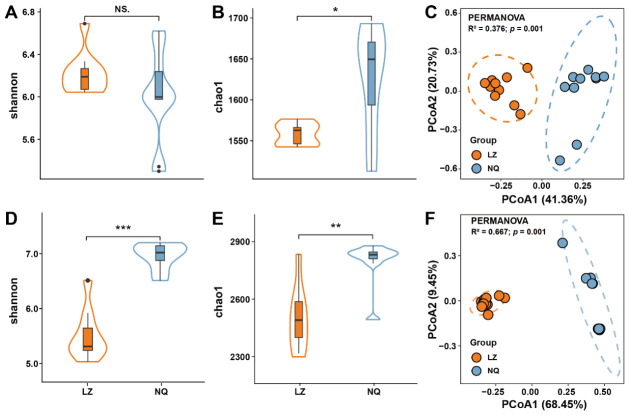
Microbial diversity and community structure in soil (*n* = 10) and water (*n* = 10). (**A**,**B**) Shannon diversity and Chao1 richness indices of soil microbial communities. (**C**) Principal coordinates analysis (PCoA) of soil microbial communities based on Bray–Curtis dissimilarity. (**D**,**E**) Shannon diversity and Chao1 richness indices of water microbial communities. (**F**) PCoA of water microbial communities based on Bray–Curtis dissimilarity. Statistical significance between the LZ and NQ groups was assessed using the Wilcoxon rank-sum test. Community dissimilarities were evaluated using PERMANOVA based on Bray–Curtis distances. NS, not significant; * *p* < 0.05; ** *p* < 0.01; *** *p* < 0.001. Solid lines in the violin plots represent the median and interquartile ranges, while dashed ellipses in the PCoA plots are used for visual clustering of the two groups and do not denote statistical confidence intervals.

To investigate how soil and water microbiomes respond to environmental variation across high-altitude habitats, 16S rRNA gene amplicon sequencing was performed on all samples. A total of 3,328,668 and 3,261,340 high-quality sequences were obtained from soil and water samples, respectively, resulting in 1741 soil ASVs and 3808 water ASVs. Distinct diversity and compositional patterns were observed in both soil and water microbiomes across regions (Figure 1). In soil communities, alpha diversity showed limited variation between regions, with no significant difference in Shannon index (Figure 2A), while Chao1 richness was higher in the NQ region (*p* < 0.05; Figure 2B). Beta-diversity analysis further revealed a clear but moderate separation between soil communities from different regions (PERMANOVA, R^2^ = 0.376, *p* = 0.001; Figure 2C). In comparison, water microbiomes exhibited more pronounced variation in both alpha and beta diversity. Shannon and Chao1 indices were significantly higher in the NQ region (*p* < 0.001 and *p* < 0.01, respectively; Figure 2D,E), indicating increased diversity and richness. Consistently, water microbial communities showed stronger regional separation (PERMANOVA, R^2^ = 0.667, *p* = 0.001; Figure 2F), with the first principal coordinate explaining 68.45% of the total variation. Overall, both soil and water microbiomes exhibited significant regional differentiation; however, the extent of variation in diversity and community composition was greater in water microbiomes.

### 3.3. Habitat-Dependent Taxonomic Differentiation Reveals Contrasting Compositional Patterns Between Soil and Water Microbiomes

**Figure 3:**
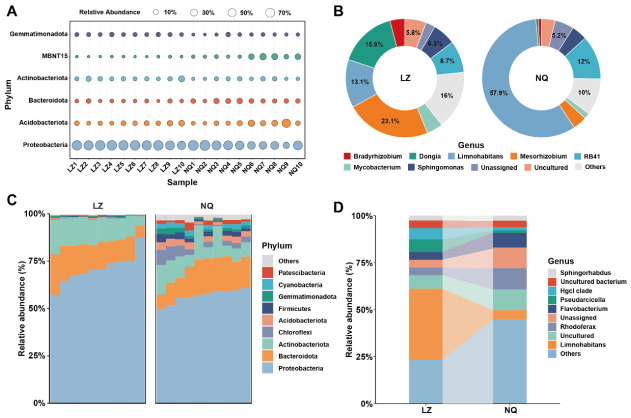
Taxonomic composition of microbial communities in soil (*n* = 10) and water (*n* = 10). (**A**) Bubble plot showing the relative abundance and prevalence of dominant bacterial phyla across all soil samples, where bubble size corresponds to relative abundance. (**B**) Donut charts representing the relative abundance of top microbial genera in soil samples from LZ and NQ regions, only categories with a relative abundance > 5% are labeled. (**C**) Bar plots showing the phylum-level taxonomic composition of water microbial communities. (**D**) Alluvial plot illustrating the shifts in genus-level community composition in water samples between the two regions.

To characterize taxonomic differences between regions, microbial communities were analyzed at both phylum and genus levels across soil and water habitats. In soil microbiomes, community composition was consistently dominated by Proteobacteria across all samples, followed by Bacteroidota and Acidobacteriota (Figure 3A), indicating a relatively conserved phylum-level structure across regions. At the genus level, however, moderate compositional shifts were observed between regions, with higher abundances of *Mesorhizobium* and *Dongia* in the LZ region and increased representation of *Limnohabitans* in the NQ region (Figure 3B), likely reflecting localized responses to subtle variations in soil organic matter and nutrient availability (Figure 1). In contrast, water microbiomes exhibited more pronounced compositional differentiation between regions. Although Proteobacteria remained the dominant phylum in both LZ and NQ, its relative abundance was higher in the LZ region, accompanied by reduced contributions from minor phyla (Figure 3C). This pronounced taxonomic restructuring coincides with the profound physicochemical divergence in aquatic habitats, where all measured parameters—pH, total nitrogen, total organic carbon, and chemical oxygen demand—varied significantly between sites (*p* < 0.001; Figure 1). At the genus level, LZ samples were strongly dominated by *Limnohabitans*, whereas NQ samples displayed a more heterogeneous community composition characterized by increased relative abundances of *Flavobacterium*, *Rhodoferax*, and uncultured taxa (Figure 3D). Overall, both soil and water microbiomes exhibited taxonomic variation between regions, but water communities showed greater shifts in dominance patterns and higher compositional heterogeneity compared with soil microbiomes.

### 3.4. Regional Differentiation Shapes Conserved Soil Microbiomes but Enhances Compositional Turnover and Interaction Complexity in Water Microbiomes

**Figure 4:**
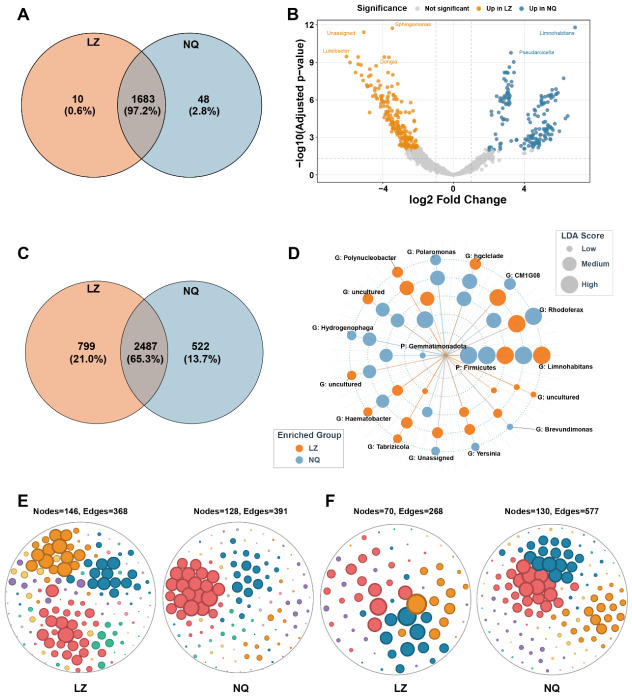
Differential taxonomic abundance and co-occurrence network analysis of soil (*n* = 10) and water (*n* = 10) microbiomes. (**A**) Venn diagram of shared and unique ASVs in soil. (**B**) Volcano plot identifying differentially abundant genera in soil between LZ and NQ regions. (**C**) Venn diagram of shared and unique ASVs in water. (**D**) LEfSe analysis identifying discriminant taxa in water, with circle size representing LDA score. (**E**) Co-occurrence networks of soil microbial communities in LZ and NQ. (**F**) Co-occurrence networks of water microbial communities in LZ and NQ. The nodes represent microbial taxa, and their colors indicate different co-occurrence modules identified within the network. Node size is proportional to the degree of connectivity.

To identify key taxonomic biomarkers and characterize microbial interaction patterns associated with the LZ and NQ regions, we combined differential abundance analysis with co-occurrence network analysis for both soil and water microbiomes. This approach accounts for the fact that microbial community assembly is influenced not only by taxonomic composition but also by complex ecological relationships among community members. In soil microbiomes, a high proportion of ASVs were shared between regions (97.2%), indicating a largely conserved core community across habitats. Nevertheless, differential abundance analysis identified a subset of taxa showing significant regional enrichment (Figure 4A,B), with *Luteibacter* and *Dongia* being more abundant in the LZ region, and *Limnohabitans* and *Pseudarcicella* enriched in the NQ region. Network analysis further revealed moderate differences in soil microbial interaction patterns between regions. The LZ soil network contained a slightly higher number of nodes (146) than the NQ network (128), suggesting limited variation in interaction complexity across regions (Figure 4E). In contrast, water microbiomes exhibited stronger regional differentiation at both compositional and interaction levels. The proportion of shared ASVs between LZ and NQ decreased to 65.3% (Figure 4C), and LEfSe analysis identified distinct regional biomarkers, including *Polynucleobacter* and *Limnohabitans* in LZ, and *Hydrogenophaga* and *Rhodoferax* in NQ (Figure 4D). Consistently, co-occurrence network analysis revealed a marked increase in interaction complexity in the NQ water microbiome, with higher numbers of nodes (130) and edges (577) compared with LZ (Figure 4F), indicating stronger restructuring of microbial interaction networks under regional differentiation. Overall, soil microbiomes were characterized by a conserved core community with moderate taxonomic and network variation, whereas water microbiomes showed greater compositional turnover and more pronounced shifts in interaction complexity across high-altitude regions.

### 3.5. Distinct Assembly Processes Govern Soil and Water Microbiomes Across High-Altitude Habitats

**Figure 5:**
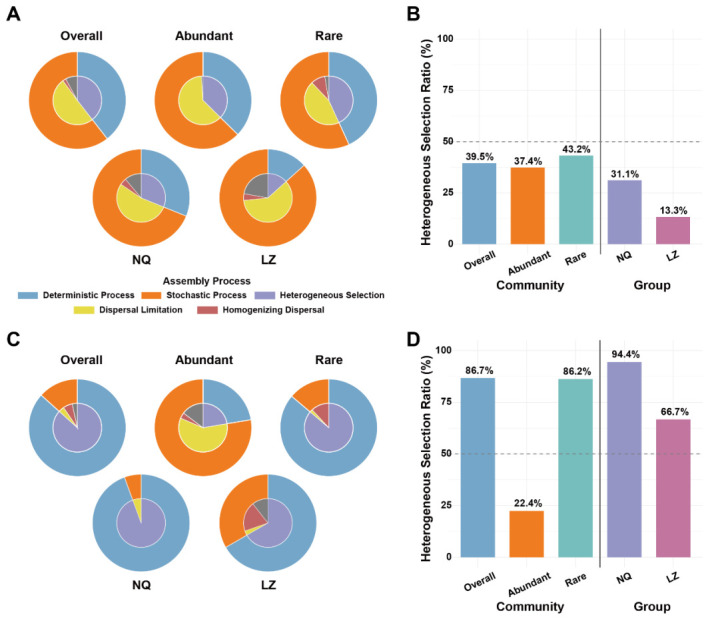
Ecological assembly processes of microbial communities in soil (*n* = 10) and water (*n* = 10). (**A**) Contributions of different assembly processes (deterministic vs. stochastic) in soil across overall, abundant, and rare taxa. (**B**) Ratio of heterogeneous selection in soil microbial communities across different taxonomic groups and regions. (**C**) Contributions of assembly processes in water communities. (**D**) Ratio of heterogeneous selection in water microbial communities. Deterministic processes include heterogeneous selection and homogenizing dispersal, while stochastic processes include dispersal limitation and undominated processes.

To quantify the ecological processes governing microbial community assembly, we evaluated the relative contributions of stochastic and deterministic processes in soil and water microbiomes. In soil communities, stochastic processes dominated across entire, abundant, and rare sub-communities in both LZ and NQ regions, consistently accounting for more than 50% of assembly processes (Figure 5A). The contribution of deterministic processes, particularly heterogeneous selection, remained comparatively low (Figure 5B), suggesting a relatively weak role of environmental filtering in shaping soil microbial communities. In contrast, water microbiomes were primarily structured by deterministic processes, which accounted for over 80% of community assembly (Figure 5C). Within deterministic processes, heterogeneous selection was the dominant component, accounting for 86.7% of the community and reaching 94.4% in the NQ region (Figure 5D), indicating stronger environmental selection in water habitats. Overall, microbial community assembly exhibited a clear habitat-dependent pattern, with stochastic processes dominating soil microbiomes, whereas water microbiomes were predominantly shaped by deterministic selection.

### 3.6. Habitat-Dependent Functional Divergence of Soil and Water Microbiomes Across High-Altitude Regions

**Figure 6:**
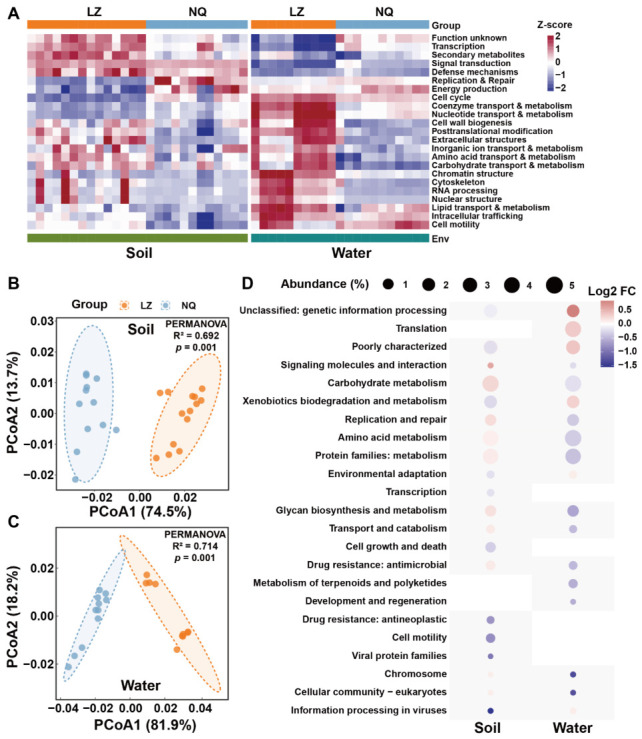
Functional profiles of microbial communities in soil (LZ, *n* = 14; NQ, *n* = 12) and water (LZ, *n* = 10; NQ, *n* = 11). (**A**) Heatmap showing the Z-score normalized relative abundance of predicted functional categories (COG) in soil and water communities. (**B**,**C**) PCoA based on Bray–Curtis distance illustrating the functional structure divergence between LZ and NQ regions for soil and water, respectively. (**D**) Dot plot displaying the Log2 fold change (FC) of functional pathways between NQ and LZ groups; bubble size represents the relative abundance of each functional category.

Building upon our 16S rRNA gene-based findings, which revealed distinct patterns of community assembly and taxonomic turnover in soil and water microbiomes across high-altitude regions, we further employed metagenomic sequencing to investigate functional responses at the ecosystem level. Heatmap analysis of functional pathways (Figure 6A) revealed clear habitat- and region-associated clustering patterns in both soil and water microbiomes.

In soil microbiomes, the LZ region showed enrichment of carbohydrate metabolism, amino acid metabolism, and inorganic ion transport and metabolism, which may reflect a metabolic strategy associated with higher nutrient availability, favoring enhanced resource utilization and metabolic activity under more favorable edaphic conditions compared with the NQ region. Conversely, the NQ region showed higher relative abundance of pathways involved in replication and repair, defense mechanisms, and signal transduction; this upregulation likely represents an enhanced physiological capacity to mitigate DNA damage and cellular stress induced by the more extreme high-altitude environmental pressures prevalent in the NQ region.

Similarly, water microbiomes exhibited pronounced regional functional variation. LZ water samples were enriched in cell wall biogenesis, as well as coenzyme, nucleotide, and lipid transport and metabolism, suggesting an investment in cellular structural maintenance. In contrast, NQ samples showed higher representation of transcription, replication and repair, and energy production-related pathways. This functional shift suggests that aquatic microbial communities in the NQ region prioritize active metabolic and genomic maintenance to sustain survival under greater environmental instability.

Principal coordinates analysis (PCoA) (Figure 6B,C) supported these observations, revealing significant regional separation in both habitats. Soil functional profiles showed clear differentiation between LZ and NQ (R^2^ = 0.692, *p* = 0.001), while water microbiomes exhibited greater divergence (R^2^ = 0.714, *p* = 0.001), with stronger ordination separation along the first axis (81.9%). Differential functional analysis (Figure 6D) further highlighted that while soil microbiomes maintained a relatively conserved functional framework across regions, water microbiomes underwent broader functional redistribution, particularly in pathways linked to environmental adaptation and xenobiotic degradation. Collectively, these data demonstrate that high-altitude soil and water microbiomes adopt distinct ecosystem-level adaptive strategies, with aquatic communities exhibiting more pronounced functional restructuring in response to regional environmental variation.

### 3.7. Habitat-Dependent Resistome Variation and Shared Antibiotic Resistance Gene Pools in Soil and Water Microbiomes

**Figure 7:**
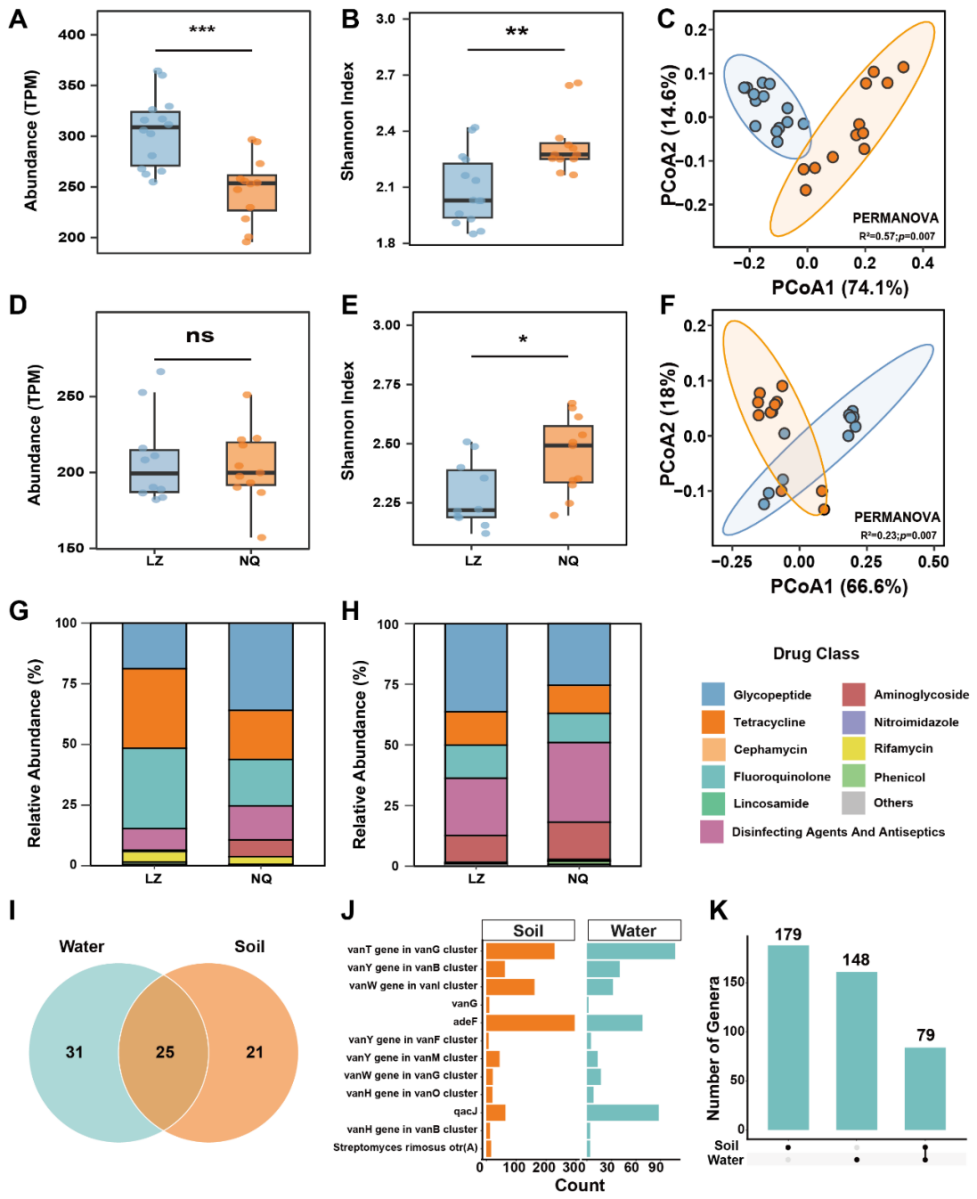
Habitat-specific resistome patterns and dissemination potential in soil (LZ, *n* = 14; NQ, *n* = 12) and water (LZ, *n* = 10; NQ, *n* = 11) microbial communities. (**A**–**C**) Abundance, Shannon diversity, and PCoA of ARGs in soil. (**D**–**F**) Abundance, Shannon diversity, and PCoA of ARGs in water. (**G**) Relative abundance of dominant ARG categories in soil. (**H**) Relative abundance of dominant ARG categories in water. (**I**) Venn diagram showing the overlap of ARG categories between soil and water. (**J**) Distribution of key ARG subtypes in soil and water. (**K**) Number of genera carrying ARGs across different habitat combinations. In panels (**A**–**F**), the color scheme is consistent: blue indicates the LZ group and orange indicates the NQ group**.** Solid and dashed lines in the plots are for visual clarity and do not represent specific statistical intervals. *, *p* < 0.05; **, *p* < 0.01; ***, *p* < 0.001; ns, not significant.

Given the ecological importance of antibiotic resistance genes (ARGs) and increasing concern over their environmental persistence, we further investigated the distribution patterns of resistomes across soil and water habitats. Resistome analysis revealed distinct habitat-dependent patterns between soil and water microbiomes (Figure 7). In soil, the LZ region showed significantly higher ARG abundance (*p* < 0.001) and lower microbial diversity (*p* < 0.01) compared with the NQ region (Figure 7A,B), with clear compositional separation observed in PCoA analysis (Figure 7C). In contrast, water microbiomes exhibited no significant difference in total ARG abundance between regions (Figure 7D), although ARG diversity was higher in the NQ region (*p* < 0.05; Figure 7E), accompanied by significant compositional variation (Figure 7F).

Taxonomic profiling of ARGs revealed habitat-specific differences in dominant resistance classes (Figure 7G,H). Soil resistomes were mainly dominated by glycopeptide and tetracycline resistance genes, whereas water microbiomes showed a more balanced distribution with a higher proportion of resistance genes associated with disinfectants and antiseptics. Despite these differences, a substantial overlap of ARG categories was observed between soil and water microbiomes (25 shared categories; Figure 7I), suggesting the presence of a shared resistome pool across habitats. Moreover, several ARG subtypes, including adeF and van cluster genes, were detected in both environments (Figure 7J), alongside widespread occurrence of ARG-carrying genera across habitats (Figure 7K), indicating potential ecological connectivity of resistance determinants. Collectively, these findings indicate that soil and water microbiomes in high-altitude ecosystems harbor overlapping resistome profiles, suggesting a potential for cross-habitat exchange of antibiotic resistance genes under environmental connectivity. 

## 4. Discussion

This study provides a comparative assessment of soil and water microbiomes in two high-altitude alpine meadow regions. Our findings indicate that habitat type strongly mediates microbial responses to regional environmental variation, characterized by divergent ecological strategies. While both habitats exhibited significant spatial differentiation in taxonomic composition, functional potential, and resistome profiles, the degree of divergence was markedly higher in aquatic communities. We infer that this disparity is linked to habitat-specific environmental filtering, where the relatively stable edaphic environment buffers microbial communities against regional fluctuations, whereas the aquatic environment’s pronounced heterogeneity in physicochemical parameters (e.g., pH, TN, COD) imposes stronger deterministic selection. These observations suggest that aquatic microbiomes function under a more constrained ecological regime, resulting in rapid taxonomic and network restructuring, whereas soil microbiomes maintain structural and functional relative stability through stochasticity.

A key finding of this study is the consistently stronger regional differentiation observed in water microbiomes compared with soil communities. While water microbiomes showed pronounced divergence in both alpha- and beta-diversity, soil communities maintained a relatively conserved structure despite detectable compositional shifts. This pattern is consistent with ecological theory suggesting that soils provide a structurally complex and buffered environment that promotes microbial persistence through microscale habitat heterogeneity and resource partitioning [35,36,37]. In contrast, aquatic environments are more directly exposed to external physicochemical conditions, leading to stronger environmental filtering and more rapid community turnover [38,39]. The lower proportion of shared ASVs in water further supports the higher sensitivity of aquatic microbiomes to regional environmental variation in high-altitude ecosystems.

Differences in community composition were further reflected in microbial interaction networks. Although soil microbiomes exhibited a highly conserved core community, moderate restructuring of co-occurrence patterns was observed across regions. In contrast, water microbiomes showed substantially greater changes in both network complexity and topology, suggesting that environmental variation influences not only taxonomic composition but also potential interaction structures. However, as co-occurrence networks infer potential rather than direct interactions, these results should be interpreted with caution. Nevertheless, the convergence of compositional and network-level changes supports the notion that aquatic microbial communities undergo broader ecological reorganization under environmental variation than soil communities.

Community assembly analyses further revealed clear habitat-dependent ecological processes. Soil microbiomes were predominantly governed by stochastic processes, whereas deterministic processes, particularly heterogeneous selection, dominated water microbiome assembly. These contrasting patterns align with ecological expectations that heterogeneous and structurally complex environments may enhance stochasticity, whereas more environmentally exposed habitats are more strongly shaped by deterministic environmental filtering [40,41,42]. In alpine meadow soils, microscale heterogeneity in resources and structure may promote dispersal limitation and ecological drift, whereas aquatic habitats are more directly influenced by environmental gradients, resulting in stronger selection pressures. The consistency among taxonomic, network, and assembly results highlights the central role of habitat-specific environmental constraints in structuring microbial communities.

Taxonomic divergence was accompanied by corresponding shifts in functional potential. Metagenomic analyses revealed significant functional differentiation in both habitats, indicating that regional environmental variation influences ecosystem-level functional traits. Soil microbiomes maintained relatively stable functional profiles despite compositional changes, suggesting functional redundancy within soil communities. In contrast, water microbiomes exhibited broader functional reorganization, particularly in pathways related to environmental adaptation and metabolism. These contrasting patterns suggest that soil and water microbiomes differ not only in taxonomic responses but also in the mechanisms underlying functional stability and reconfiguration in alpine ecosystems.

Distinct habitat-dependent resistome structures were observed between soil and water microbiomes. Although both habitats harbored unique antibiotic resistance gene (ARG) profiles, a substantial proportion of ARG categories and several dominant subtypes were shared, suggesting the presence of overlapping resistome pools across terrestrial and aquatic environments. Importantly, the detection of shared ARGs does not imply active horizontal gene transfer between habitats; rather, it highlights potential ecological connectivity and common environmental reservoirs shaping ARG distribution in alpine ecosystems. Given increasing concerns regarding environmental antimicrobial resistance, future studies incorporating mobile genetic elements, plasmid characterization, and genome-resolved approaches will be essential to elucidate ARG dynamics and transmission potential across habitats.

Several limitations should be acknowledged. The logistical constraints of sampling in high-altitude environments limited spatial coverage and precluded seasonal replication. As a result, caution is warranted when extrapolating these findings beyond the studied regions of the Qinghai–Tibetan Plateau. In addition, this study relied on community-level sequencing approaches, which cannot directly resolve microbial activity or confirm ecological interactions and ARG transfer events. Future studies integrating multi-omics approaches, environmental measurements, and genome-resolved analyses will provide deeper insights into microbial ecological processes in alpine ecosystems.

In conclusion, this study demonstrates that soil and water microbiomes exhibit distinct ecological responses to regional environmental variation in high-altitude alpine meadow ecosystems on the Qinghai–Tibetan Plateau. Soil communities are characterized by a conserved core microbiome and predominantly stochastic assembly processes, whereas water microbiomes show stronger taxonomic turnover, more pronounced network reorganization, greater deterministic selection, and broader functional restructuring. Despite these differences, both habitats share overlapping resistome components, suggesting ecological connectivity between terrestrial and aquatic microbial systems. These findings enhance our understanding of how habitat characteristics shape microbial diversity, ecological interactions, functional traits, and antibiotic resistance patterns in alpine ecosystems.

## 5. Conclusions

In summary, this study reveals distinct ecological responses of soil and water microbiomes to regional environmental variation in high-altitude alpine meadow ecosystems on the Qinghai–Tibetan Plateau. Although both habitats exhibited significant shifts in community composition, functional potential, and resistome structure, the magnitude and ecological mechanisms underlying these responses differed markedly between soil and water environments. Soil microbiomes were characterized by a conserved core community and predominantly stochastic assembly processes, whereas water microbiomes showed stronger taxonomic turnover, more complex interaction networks, greater deterministic selection, and broader functional reorganization. Collectively, these findings demonstrate that habitat type is a key determinant of microbial ecological strategies in alpine ecosystems, shaping how microbial communities respond to environmental heterogeneity across taxonomic, functional, and resistome dimensions. These habitat-specific responses suggest that soil and aquatic microbial systems may contribute differently to ecosystem-level processes under environmental change, and should therefore be considered separately in the monitoring and management of alpine meadow ecosystems.

## Figures and Tables

**Table 1:** Geographic characteristics of sampling sites.

Region	Longitude (°E)	Latitude (°N)	Approx. Mean Altitude (m)
Qinglong, Nagqu	90.6688	31.1625	~4700
Pubao, Nagqu	90.1534	31.3726	~4700
Deqing, Nagqu	90.1497	30.5935	~4700
Mirui, Nyingchi	94.5288	29.4603	~2900
Mirui, Nyingchi	94.3141	29.2717	~2900
Mirui, Nyingchi	94.4215	29.3662	~2900

## Data Availability

The original contributions presented in this study are included in the article. Further inquiries can be directed to the corresponding author.

## Abbreviations

The following abbreviations are used in this manuscript:

ARG	Antibiotic resistance gene
ASV	Amplicon sequence variant
CARD	Comprehensive antibiotic resistance database
COG	Clusters of orthologous groups of proteins
GO	Gene ontology
KEGG	Kyoto encyclopedia of genes and genomes
LDA	Linear discriminant analysis
PCR	Polymerase chain reaction
QTP	Qinghai–Tibetan Plateau
TPM	Transcripts per million
